# Infliximab as *ultima ratio* in a case of refractory non-Crohn’s disease complex fistula system: case report and mini-review of the literature

**DOI:** 10.3389/fgstr.2026.1815049

**Published:** 2026-06-16

**Authors:** Nada Agbariah, Matthias Kis, Christine Manser, Luc Biedermann, Gerhard Rogler

**Affiliations:** 1 Department of Gastroenterology and Hepatology, University Hospital of Zurich, Zurich, Switzerland; 2 Department of Gastroenterology, Kantonsspital Frauenfeld, Frauenfeld, Switzerland

**Keywords:** abscess, anti-TNF, fistula, infliximab, non-Crohn’s fistula

## Abstract

Infliximab (IFX) has been a first-line treatment for perianal fistulizing Crohn’s disease (CD) since 1999. However, its role in non-CD-related fistulas remains largely unknown, despite the established involvement of tumor necrosis factor (TNF) in both types of fistulas. A 76-year-old man with a history of prostate cancer presented in 2024 with recurrent fistulas and a presacral wound cavity with sepsis. He had been hospitalized approximately once a month over the past year due to fever and uncontrolled infection. The patient was initially treated with radiation in 2017. Despite this treatment, he developed increasing polyuria related to prostatic hyperplasia, which led to his first operation (prostatectomy) with accidental rectal injury, followed by multiple surgeries for complications and subsequent abscess formation. Histologically, there was no evidence of CD. Due to a lack of surgical options and failed endoscopic treatments, the patient was considered for exploratory IFX therapy. The patient received IFX induction therapy (5 mg/kg every 2 weeks for the first two doses) in May 2024, followed by maintenance therapy (5 mg/kg every 8 weeks). Within just 2 weeks, he reported the first significant clinical improvement. After 22 weeks of therapy with IFX, he reported feeling well, with no abdominal pain and a weight gain of 6 kg. At the 1-year follow-up, no further hospitalizations due to uncontrolled presacral infection with fever and anal pus discharge were reported, and the presacral wound cavity was found to be reduced on MRI from 37 mm × 43 mm × 62 mm (March 2024) to 23 mm × 22 mm × 45 mm (May 2025). Despite a history of recurrent urinary tract infections (UTIs) associated with mono-J catheters, the frequency of UTIs did not increase during therapy with IFX. This case indicates that infliximab may represent an effective therapeutic option for non-CD-related fistulas when there are no other therapeutic options available.

## Introduction

Infliximab (IFX) has been a first-line treatment for perianal fistulizing Crohn’s disease (CD) since 1999, as endorsed by the European Crohn and Colitis Organization (ECCO) and the American Gastroenterological Association (AGA). However, its role in non-CD-related fistulas remains largely unknown, despite the established involvement of tumor necrosis factor (TNF) in both types of fistulas.

IFX is a chimeric monoclonal antibody (75% human and 25% murine) that binds TNF (1). TNF is mainly produced by monocytes and macrophages (among many other cells) and activates T cells as well as other immune cells and promotes inflammation (2). Its expression is upregulated in patients with Crohn’s disease (CD) and in CD-related fistula (CDF), especially in those CD patients with perianal disease, where TNF levels in the blood and tissue correlate well with the Perianal Activity Index (PAI) (3, 4). The first study demonstrating the efficacy of IFX in reducing fistula activity in CD patients was published in 1999 by Present and colleagues (5). Further data collected between 1999 and 2010 confirmed the role of IFX in closing perianal CD fistulas, with 108 of 156 patients achieving complete closure of at least one fistula after a median of 1 year (6). IFX therapy usually requires long-term maintenance treatment rather than a single dose to be effective in perianal Crohn’s disease (PACD) (7). Today, IFX is an established treatment for PACD, with European Crohn and Colitis Organization (ECCO) and American Gastroenterological Association (AGA) guidelines recommending it for induction and maintenance of remission in complex perianal fistulas (8, 9). Recent studies highlight the need for higher induction and maintenance IFX trough levels in PACD (10–12), a finding reinforced by the ATLANTIC study presented at ECCO 2025 (13), suggesting distinct pharmacokinetics in this population.

For non-CD-associated fistulas, standard treatment remains primarily surgical or endoscopic. Surgical options include fistulectomy, marsupialization, setons (partial sphincter-preserving), advancement flaps, and ligation of the intersphincteric fistula tract (sphincter-preserving) (14). Endoscopic treatments mainly comprise over-the-scope clips and endoscopic vacuum therapy although evidence is limited to case reports and series (15–17). Combined surgical and endoscopic approaches have also been reported (18, 19). In contrast to PACD, no established medical therapies exist for non-CD perianal fistulas. Limited literature reports rare cases of non-CD fistulas responding to infliximab; for example, three cases of enterocutaneous fistulas treated successfully with a single infliximab dose nearly 20 years ago (20), and a recent case of radiation-induced vesicovaginal fistula treated with long-term IFX (21).

## Case presentation

A 76-year-old male patient with prostate adenocarcinoma underwent radiation therapy in 2017. Due to persistent polyuria related to prostate hyperplasia with up to 30 micturitions per day, the patient underwent a prostatectomy in October 2018, which was complicated by an accidental rectal injury, necessitating a Hartmann procedure with sigmoidostomy. In October 2018, surgery was performed for drainage of a fistulizing abscess between the symphysis and prostatic urethra. Because of an abdominal wall necrosis, the Hartmann procedure was reversed in December 2018, with a descendostomy, rectus abdominis muscle flap placement, and bilateral mono-J catheter insertion.

An abscess and fistula system developed in the lower abdominal wall, requiring surgical drainage in February 2019. Throughout 2019, multiple surgeries were performed for drainage of intra-abdominal abscesses. Due to recurrent urinary tract infections (UTIs), long-term antibiotics were administered from 2019 to 2022.

Endoscopic vacuum therapy was attempted twice—February 2022 and December 2023 to February 2024—with good temporary result (**Figure 1**). Due to the many adhesions, no surgical options were available anymore. The abscesses and infections became uncontrollable, and the patient was hospitalized frequently and transferred to our university hospital in March 2024 for re-evaluation.

**Figure 1:**
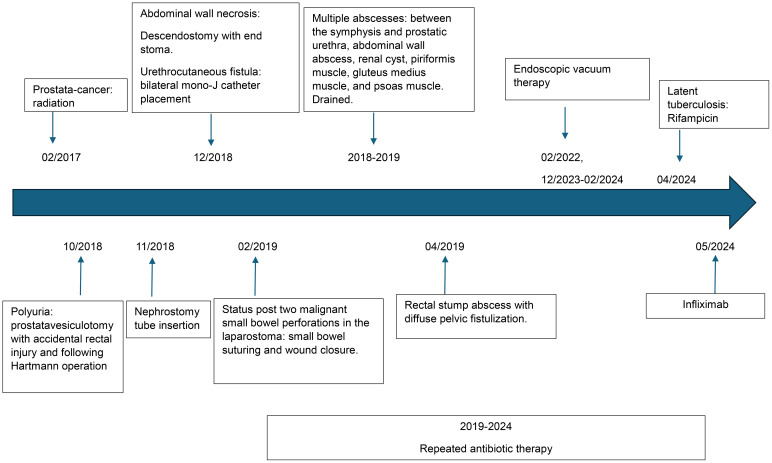
Therapy timeline.

At presentation, the patient showed a complex fistula system. Histopathology of the colon and rectal resection specimens from previous operations showed no typical features of inflammatory bowel disease or malignancy. Specifically, in the resection specimens of the colon and rectum and of a recto-urethral fistula, there were features of acute serositis, mixed acute and chronic inflammation, and necrotic adipose tissue. Additionally, the clinical picture argued against isolated perianal CD, for which IFX is commonly used. The fistula was considered likely to be iatrogenic, as the prostatectomy surgical report documented an accidental rectal injury. Two months later, the patient presented with abscesses and a fistula system (**Figure 1**). Nonetheless, it should be acknowledged that occult Crohn’s-like fistulizing disease cannot be entirely excluded based on negative resection histology alone, especially in a complex postsurgical and irradiated pelvis.

At presentation in March 2024, a rectosigmoidoscopy revealed a rectal wall defect communicating with the small bowel (**Figure 2**). MRI showed a presacral abscess with an enteroenteric rectum–ileum fistula (**Figure 3**). Clinically, the patient had fever, elevated CRP and leukocytes, and a purulent anal discharge.

**Figure 2:**
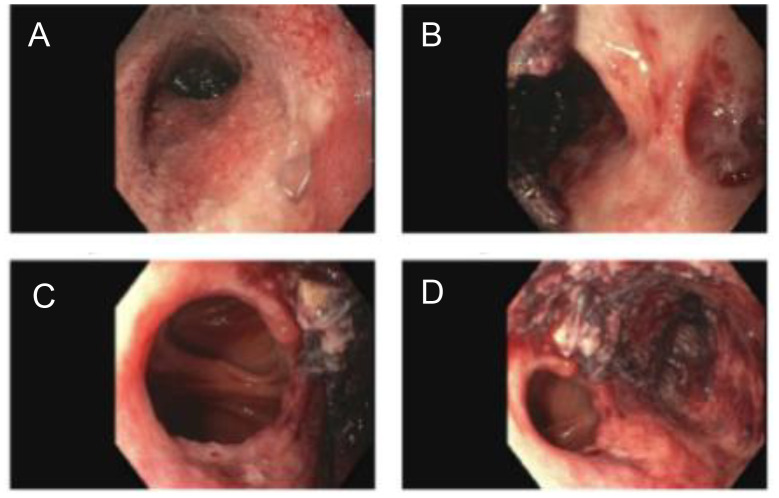
Endoscopy before IFX therapy. **(A)** granulation tissue. **(B)** small site defect. **(C, D)** connection to the small bowel.

**Figure 3:**
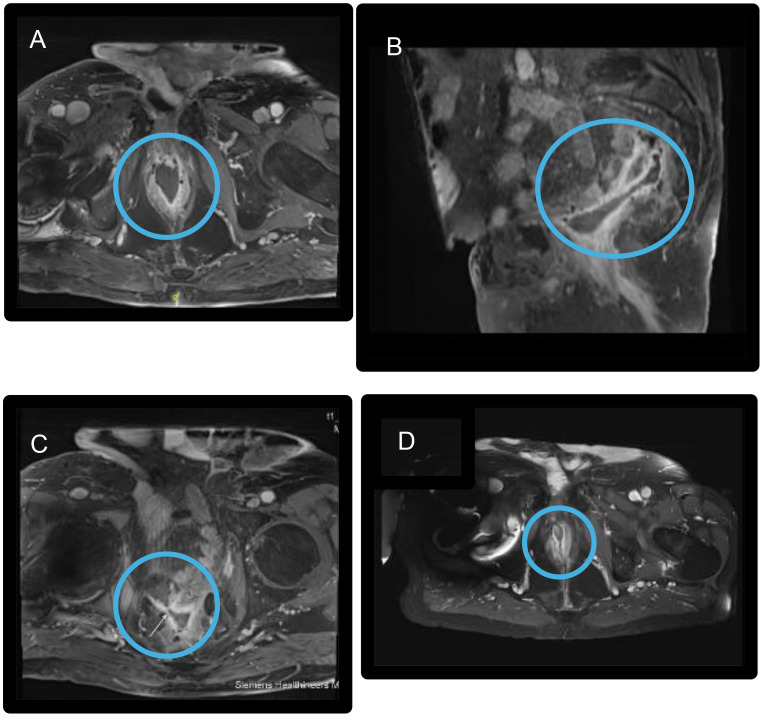
Comparison MRI. **(A)** Transversal MRI before IFX. **(B)** Sagittal MRI before IFX. **(C)** Possible fistula before IFX. **(D)** Transversal MRI after IFX.

Given the failure of endoscopic therapy and the lack of surgical options, medical therapy was considered. Analogous to the standard of care for IBD, we considered anti-TNF-alpha after failure of surgical therapy (22). In fact, the use of anti-TNF in PACD is supported by TNF’s central role in inflammation (23, 24) as well as the above-mentioned positive case reports of anti-TNF in non-Crohn’s fistula (20, 21). The patient’s comorbidities included atrial fibrillation, rheumatoid arthritis, compensated hypertensive heart disease, and prediabetes. The patient’s rheumatoid arthritis was asymptomatic with a maintenance therapy of prednisone 5 mg/day. Given the presence of cardiac comorbidities, adalimumab was excluded. There were no contraindications for infliximab. The previous prostate cancer diagnosis was fully treated with radiation and prostatectomy, so it did not represent a contraindication to infliximab, consistent with current evidence suggesting an acceptable safety profile of anti-TNF agents in patients with a history of prior malignancy (25, 26). Because of the bilateral mono-J catheter insertion, there was an increased pre-existing risk for UTI. We discussed this with the patient and his family, and it was decided to proceed with the medication, monitoring for a potential increase in UTIs, and eventually considering a parallel antibiotic prophylaxis. Screening was negative for HIV, HCV, and HBV but revealed latent tuberculosis. Rifampicin therapy was started 4 weeks before initiating IFX and was continued for a total of 4 months. The patient and his family were well-informed about the off-label use of infliximab in this case and were eager to proceed.

## Outcome and follow-up

Induction therapy with IFX (5 mg/kg every 2 weeks for two doses) began in May 2024, followed by maintenance dosing every 8 weeks. Within 2 weeks, the patient reported initial subjective improvements in wellbeing and clinical condition. At 22 weeks, he reported a notably increased appetite with a weight gain of 6 kg and no abdominal pain, fever, or anal pus discharge anymore. At 1 year, he had no hospitalizations related to the presacral wound cavity infection, remained clinically stable and physically active, and resumed usual activities. MRI showed that the presacral wound cavity reduced from 37 mm × 43mm × 62 mm in March 2024 to 23 mm × 22 mm × 45 mm in May 2025. The IFX trough level was 8.2 µg/mL, with no detectable anti-IFX antibodies.

Despite IFX’s known infection risk (24), the patient tolerated the therapy very well. Previously experiencing up to 10 UTIs per year, mostly related to mono-J catheters, he had only two UTI episodes during the first treatment year, both treated with antibiotics. Thus, under IFX treatment, the frequency of UTIs did not increase, even in this high-risk situation.

## Discussion

IFX therapy was associated with marked clinical improvement in this case of non-CD fistula. The patient experienced significant quality-of-life improvement, with reduced hospitalizations and enhanced general wellbeing. Radiologically, the presacral abscess size decreased notably, and clinically, the purulent anal discharge stopped.

We consider the patient’s clinical improvement to be mainly attributable to infliximab. As mentioned above, the role of TNF in CD fistulas is nowadays well known, and an increased production of TNF can also be postulated in non-Crohn’s fistula. For years, our patient had been repeatedly treated with antibiotics and endoscopic vacuum therapy, as shown in **Figure 1**; however, none of these treatments provided long-term benefit. The initiation of anti-TNF therapy represented a turning point in his clinical course. Nevertheless, the synergistic effect of the combined treatments cannot be fully excluded.

Unlike previously reported cases of non-CD fistulas treated with a single IFX dose, our patient had a chronic, complex fistula system requiring ongoing therapy, in the sense of a classical maintenance treatment for a chronic inflammatory condition driven by upregulated local immune response. Based on that rationale and in view of the highly encouraging clinical course of disease in this exceedingly difficult-to-treat scenario, the following conclusions appear reasonable: Firstly, ongoing maintenance treatment will be pursued, ideally under pro-active trough-level monitoring, equivalent to our preferred approach in severe penetrating perianal CD. Secondly, despite this *n*-of-1 sample size and the limited data on the potential of IFX on non-CD fistula in the prior literature—and acknowledging that dedicated trials to evaluate this approach are unlikely to be conducted—we suggest considering anti-TNF therapy for refractory and severe non-CD fistulizing disease when no other therapeutic options are available.

## Conclusions

Our experience reported here, in conjunction with the scarce promising available literature, suggests that IFX may be a valuable *ultima ratio* for patients with complex perianal (and perhaps also non-perianal) fistula systems when surgical and endoscopic options are exhausted. The nature of this case report should be considered exploratory and limited to a single case, for which no further standard therapy options were available. The patient and his family were motivated to try an off-label therapy to reduce the number of hospitalizations. Randomized prospective studies are needed to investigate, on a larger scale, the effect of infliximab in non-Crohn’s fistulas.

## Data Availability

The original contributions presented in the study are included in the article/supplementary material. Further inquiries can be directed to the corresponding author.
